# The Health and Working Conditions of Women Employed in Child Care

**DOI:** 10.3390/ijerph14030283

**Published:** 2017-03-09

**Authors:** Laura Linnan, Gabriela Arandia, Lori A. Bateman, Amber Vaughn, Natalie Smith, Dianne Ward

**Affiliations:** 1 Department of Health Behavior, Gillings School of Global Public Health, University of North Carolina at Chapel Hill, Chapel Hill, NC 27599, USA; arandia@live.unc.edu; 2 Center for Health Promotion and Disease Prevention, University of North Carolina at Chapel Hill, Chapel Hill, NC 27599, USA; lbateman@email.unc.edu (L.A.B.); avaughn@email.unc.edu (A.V.); dsward@email.unc.edu (D.W.); 3 Department of Biostatistics, Gillings School of Global Public Health, University of North Carolina at Chapel Hill, Chapel Hill, NC 27599, USA; natsmith@live.unc.edu; 4 Department of Nutrition, Gillings School of Global Public Health, University of North Carolina at Chapel Hill, Chapel Hill, NC 27599, USA

**Keywords:** low wage workers, child care workers, work conditions, job strain

## Abstract

Over one million women are employed in child care and are among the lowest wage workers in the US. The health and working conditions of 674 child care workers (118 administrators and 556 staff) from 74 centers is described using baseline data from a larger intervention trial. Participants were 39.9 (±13.0) years old; 55.4% African American, 37.1% Caucasian, and 5.3% of Hispanic ethnicity. Seventy-six percent reported having an Associate’s degree or less; 42% were classified as at or below poverty (<$20,000); and exhibited many health risks such as excess weight, insufficient activity, poor diet, and inadequate sleep. We investigated potential differences by income and job category. Lower income participants were significantly more likely to be current smokers (19.9% vs. 11.7%), drink more sweetened beverages (1.9 vs. 1.5), and report higher depressive symptoms (15.5 vs. 12.6). Administrators worked more hours weekly compared to staff (46.4 vs. 40.6), are less active (100 vs. 126 min/week), more sedentary (501 vs. 477 min/day), and reported higher job demands (13.3 vs. 12.5). Given the numerous health issues and challenging work conditions, we hope our results serve as a call to action for addressing low wages and the work environment as a means of influencing the health and well-being of child care workers.

## 1. Introduction

There are more than 1.3 million child care workers in the U.S. [1] of which 95.5% are women [2]. In fact, child care is one of the 10 most prevalent occupations for women [2]. While they are entrusted with our most valuable asset, our children, child care workers are among the lowest paid occupations, often having earnings below poverty level [3,4]. The 2016 Occupation Outlook Handbook reports that the average pay for child care workers in the U.S. is $9.77 per hour or $20,320 annually [1]. As low wage workers, they likely face higher rates of chronic disease and shorter life expectancies [5,6,7]. Furthermore, low wage workers often experience challenging working conditions as demonstrated in a review by Landsbergis and colleagues who found that low wage workers were more likely to experience higher job insecurity and job strain than higher wage workers [8].

Very little information is available about the health status of child care workers. The largest study with more than 2000 female child care workers in Pennsylvania found that 75% had one or more chronic health conditions and 20% had three or more conditions [9]. However, the sample was limited to employees at Head Start, a federally-sponsored program that provides comprehensive early childhood education, health, nutrition, and parent engagement services to low-income children and their families. While Head Start programs have more standardized organizational structure and financial support compared to center-based programs, the study suggests that many of the workers were still struggling with multiple health issues. Among the few other studies with child care workers, results suggest elevated levels of emotional distress [10], multiple health risk behaviors [11], and increased prevalence of overweight and obesity [12].

Working conditions such as long hours, high job demands, low wages, lack of health benefits, and high turn-over may be impacting the health of child care workers. A recent review by Cumming on child care workers’ well-being identified 30 studies conducted in the U.S. and abroad that help document these challenging working conditions, several of which also demonstrated how these work conditions are related to adverse effects on workers’ psychological and emotional health [13]. Other studies have shown that the working conditions of the child care job can place workers at risk for infectious diseases, injuries, and other occupational hazards [14,15,16]. Unfortunately, very few studies have examined the relationship between working conditions and workers’ physical and emotional health status and/or health behaviors.

The purpose of this study is to describe the health status, health behaviors, and working conditions of child care workers and to explore how income and job position may be associated. We believe considering working conditions will address an important gap in the literature about the health of child care workers. We will also discuss the implications of these results for both practice and research that might improve the health and working conditions for child care workers in the future.

## 2. Materials and Methods

This study used baseline data from a group randomized control trial (ClinicalTrials.gov registration number NCT02381938) conducted in North Carolina to evaluate the impact of a child care-based worksite wellness intervention. For the purpose of this paper, we used baseline data only, collected prior to randomization and intervention implementation. All protocols were approved by the Institutional Review Board at the University of North Carolina at Chapel Hill (#13-2438).

### 2.1. Sample Recruitment and Eligibility

A convenience sample of child care centers and their workers, both administrators and staff, were recruited to join a group randomized control research trial using a multi-phase recruitment strategy. We targeted counties in North Carolina where we had existing community partners that could help facilitate an introduction to local child care centers. Moreover, we targeted geographical areas that had sufficient numbers of medium to large centers to ensure that we would reach recruitment goals. Across four waves, we recruited seven counties that represented a combination of rural, suburban, and urban area, moderate to low income, and similar racial and ethnic populations compared to the state. Centers in these counties were identified from an online database of licensed child care programs available through the North Carolina Division of Child Development and Early Education [17]. Following introductions from the community partners, the research team initiated direct contact with local child care centers via mail/email letters of invitation and follow up telephone calls. During calls, research assistants confirmed center eligibility, reviewed study details, and assessed center interest. Eligible centers had to be in business for at least two years with no plans to close in the following 18 months and have at least four employees (one administrator and three additional staff) willing to participate. Once center eligibility was confirmed, research assistants met with interested staff in-person to confirm eligibility, review study details, and collect informed consent. Eligible staff had to be at least 18 years old, able to read and speak English, and pass a physical activity readiness screening (PAR-Q+ [18]) (and obtain medical approval if required).

### 2.2. Data Collection and Measures

Participants completed several measures, including physical assessments, physical activity monitoring, and web-based and paper surveys. Data were collected primarily during an onsite visit by data collectors who were trained and certified on all protocols. Specific measures are described below:

*Physical Assessments*. Height, weight, waist circumference, heart rate, and blood pressure were assessed using standard protocols [19,20]. Height was measured to the nearest 1/8 inch with a Shorr measuring board (Shorr Productions, Olney, MD, USA). Weight was measured to the nearest 0.1 pound with a Seca model 874 portable electronic scale (Seca Corporation, Columbia, MD, USA). Height and weight measures were taken with shoes and heavy clothing removed. Waist circumference was measured with a Gulick II (Patterson Medical, Warrenville, IL, USA) measuring tape to the nearest 0.1 cm at the level of the iliac crest. Heart rate and blood pressure were assessed using a digital blood pressure monitor (Omron, Kyoto, Japan) while participants sat quietly with their legs uncrossed and feet flat on the floor. All measures were taken twice. If the two measurements were within reasonable agreement (≤¼ inch for height, ≤1.0 lb. for weight, ≤3.0 cm for waist circumference, and <5 mmHg for systolic and diastolic blood pressure) the final measure was recorded as the average of the two values. Otherwise, a third measure was taken. Height and weight were used to calculate body mass index (BMI) (weight in kg/height in m^2^) and weight status (underweight = BMI < 18.5, normal weight = BMI 18.5–24.9, overweight = BMI 25.0–29.9, obese = BMI ≥ 30.0). Systolic and diastolic blood pressure were used to determine mean arterial pressure.

*Physical Activity Monitoring*. Physical activity was assessed using one week of accelerometer monitoring using a GT3X ActiGraph monitor (ActiGraph LLC, Pensacola, FL, USA). Participants were fit with a belt that allowed the monitor to be worn over the right hip and were instructed to wear the monitor for the next seven days, 24 h a day except during water activities. Monitors were programmed to sample at 30 Hz. Data from the monitor were downloaded using ActiLife software (ActiGraph LLC). Adult-specific cut points were applied to determine minutes of sedentary activity (0–100 counts/min, <1.5 METS) and minutes of moderate-to-vigorous physical activity (MVPA) (≥2020 counts/min, 3.0 METS) [21,22] for participants with at least eight hours of wear time on a minimum of four days.

*Web-Based Surveys*. Health behaviors were assessed using a series of self-administered surveys collected via an online system known as the Carolina Health Assessment and Research Tool (CHART) [23]. More specifically, CHART assessed dietary intake, tobacco and e-cigarette use, sleep, and emotional health.

Dietary intake was assessed using items from the Dietary Screener Questionnaire [24,25] and the Diet History Questionnaire [26]. Within this survey, five items asked specifically about intake of fruits, fruit juice, potatoes, beans and legumes, and other vegetables; and two items asked about intake of sugar-sweetened and artificially-sweetened beverages. The five items related to fruits and vegetables were summed to get a total daily fruit and vegetable intake; and the two items related to beverages were summed to get a total sweetened beverage intake.

Tobacco and e-cigarette use was assessed using two items modified from the 2012 Behavioral Risk Factor Surveillance System Questionnaire (BRFSS) [27] that asks participants to estimate the average use of each during the past 30 days. Items were used to identify participants that are current smokers or had ever used e-cigarettes.

Sleep duration was assessed using one item extracted from the Pittsburgh Sleep Quality Index (PSQI) [28], which asked participants to estimate their typical hours of sleep per night during the past 30 days.

Emotional health was assessed using a combination of indices related to distress, depressive symptoms and job strain. Distress was measured using one item from the Society for Behavioral Medicine’s Common Data Elements [29] which asked participants to rate their level of distress during the past week on a scale of 0 to 10 (0 = no distress, 10 = extreme distress). Depressive symptoms were measured using the 20-item Center for Epidemiologic Studies Depression Scale (CES-D) [30]. As specified in standard scoring protocols, item responses (scored 0–3) are reverse-coded when appropriate and summed to create a total depressive symptom score (ranging from 0−60) with ≥16 indicating high risk of clinical depression. Job strain indicators were measured with 15 items derived from the Job Content Questionnaire (JCQ) [31], specifically items required to calculate job demand (scores ranging from 5−20), job control (scores ranging from 12−48), and job insecurity (0,1). These items ranged from 1 (strongly disagree) to 4 (strongly agree) and were reverse-coded when necessary. Job demands was composed of 5 items (e.g., “My job requires working very fast”) and a higher score indicates more job demands. Job control is comprised of two subscales, skill discretion (6 items, e.g., “My job requires me to learn new things”) and decision authority (3 items, e.g., “I have a lot to say about what happens at my job”) and a higher score indicates less job control. Job insecure participants answered “Strongly disagree” or “Disagree” to “My job security is good” [32].

The web-based surveys also captured demographic variables including age, sex, race, ethnicity, education, marriage status, household size, household income, and hours worked (at the enrolled childcare center plus any from a second job). Household income (assessed categorically) was used to identify participants as below poverty (<$20,000) or above poverty (≥$20,000). The below poverty cut point was based on the median household size in our sample (three) and income thresholds set in the 2016 Federal Poverty Guidelines [33] ($20,160 for a household of three). Seventy-four participants declined to answer this question, and were thus considered missing for income-related analyses.

Among these demographic items, there was also a question that asked participants to identify their role at the center (e.g., job position), with response options including owner, director, assistant director, lead teacher, assistant teacher, and other. Administrators (defined as owner, director, or assistant director) were asked additional questions about center demographics, including years of operation, hours of operation, enrollment fees, star rating, number of kids in care, number of employees, center type, current participation in the Child Adult Care Food Program (CACFP), and accreditation by the National Association for the Education of Young Children (NAEYC). CACFP is a federally-funded U.S. program that provides reimbursement for meals and snacks served in child care programs that serve low-income families. The NC’s Quality Rating and Improvement Systems (QRIS) [34] star rating is an indicator of quality based on a five-point scale, where a rating of one star corresponds to meeting minimum licensing standards and five stars represents the highest quality and voluntarily compliance with higher standards related to programming and staff education.

### 2.3. Statistical Analysis

First, we examined descriptive summary statistics (Table 1 and Table 2). Drawing from the measures described above, we examined demographic characteristics (e.g., age, sex, race and ethnicity, education), indicators of health status and behavior (e.g., weight, waist circumference, heart rate, blood pressure, diet, physical activity, cigarette and e-cigarette use, sleep, emotional health), and indicators of working conditions (e.g., hours worked, job demand, job control, job security). Next, we explored differences in health status, health behaviors, and working conditions by income status (below vs. above $20,000) and job position (administrator vs. staff). Differences were assessed using chi-square tests (categorical outcomes) or two independent sample *t*-tests (continuous outcomes) using SAS software 9.4 (SAS Institute Inc., Cary, NC, USA). Due to the high number of hypothesis tests conducted, the raw *p*-values were adjusted to control for the overall Type I error rate; however, these adjusted *p*-values did not change any conclusions about statistical significance and thus the raw *p*-values are presented for simplicity in Table 3 and Table 4. 

## 3. Results

### 3.1. Participant Demographics

While the full sample included 697 child care workers from 74 centers, we narrowed the sample to include only the 674 female workers (118 administrators and 556 staff) for this study. Demographic characteristics of these participants are presented in Table 1. On average, participants were 39.9 (±13.0) years old. The majority (55.4%) were African American, 37.1% were Caucasian, and 5.3% were of Hispanic ethnicity. The majority (76.0%) of participants attained an Associate’s degree or less. Forty two percent of participants were classified as at or below poverty (based on our cut point of <$20,000).

We explored demographic differences by job position (administrators vs. staff). Administrators were slightly older than staff (43.8 years vs. 39.1 years); were more likely to be White (45.7% vs. 35.3%); attained more education (46.6% having completed a bachelor’s degree or higher vs. 19.3% of staff); and had higher household incomes (89.1% reported an income greater than $20,000 vs. 44.7% of staff). Administrators were more likely to be married or living with a partner (63.6% vs. 47.3%). No differences were observed between administrators and staff regarding household size.

### 3.2. Child Care Center Demographics

Seventy-four child care centers were enrolled in this study, demographics for which are described in Table 2. Participating centers had a high quality rating, averaging 4.3 (±0.7) stars. Centers had, on average, 56.3 (±32.5) children enrolled and 12.1 (±8.6) staff employed. Centers reported being open for 13.1 (±3.4) h per day and were predominately privately owned (68.1%), though nearly all accepted subsidies (97.3%) and participated in CACFP (84.7%).

### 3.3. Health Indicators Overall

Participant health indicators are presented in Table 3. Participants appeared to have many indicators of poor health status and behavior. Participants’ average BMI was 34.5 (±9.0) with 22.2% of participants classified as overweight and 66.3% classified as obese. Average waist circumference was 106.5 cm (±18.2), well above the 88.0 cm cut point for women that is associated with increased risk for disease [35]. Twenty-six percent of participants had high blood pressure (defined as pressures at or above 140/90). Although we are unable to report on the use of blood pressure medication, 33.5% of our sample reported on the PARQ+ (from the eligibility screening) being told by a physician that they have high blood pressure.

Regarding health behaviors, participants accumulated an average of 122 (±104) min per week of MVPA, which is below the recommended 150 min per week [36]. Also, participants accumulated 481 (±72.4) min per day of sedentary time, roughly 8 h a day. Participants reported eating fruits and vegetables an average of 2.58 (±1.72) times per day and drank sweetened beverages on average 1.71 (±1.82) times per day. Even if participants consumed a cup each time they ate fruits or vegetables, they still consumed far below the 3.5–4.5 cups per day that is recommended for women [37]. Overall, 15.6% were current smokers, and 9.9% reported having used an e-cigarette. On average, participants reported sleeping 6.37 (±1.35) h per night, lower than the 7–8 h per night that is recommended [38]. Perceived level of distress was reported as 4.02 (±2.78) and CES-D depression score was 13.9 (±9.17) with 36.1% reporting scores at or above 16 (the criteria for clinical depression).

### 3.4. Health Indicators by Income and Job Position

Significant differences were noted between participants making below $20,000 compared to those with an income above $20,000. Specifically, lower income participants were significantly more likely to be current smokers (19.9% vs. 11.7%, *p* = 0.0057), drink more sugar-sweetened beverages on a daily basis (1.9 vs. 1.5, *p* = 0.0057), and report higher depressive symptoms (15.5 vs. 12.6, *p* = 0.0002). A higher percentage of lower income participants were classified as at or above the typical cutoff of 16 (41.6% vs. 31.3%, *p* = 0.0094). The lowest income participants (below $20,000) were also less sedentary (468 min/day vs. 491 min/day, *p* = 0.0006). There were few differences in health indicators by job position; however, compared with staff, administrators accumulated an average of 26 min less of MVPA per week (126 vs. 100 min/week, *p* = 0.0048) and 24 more minutes of sedentary time per day (477 vs. 501 min/day, *p* = 0.0051).

### 3.5. Indicators of Working Conditions Overall

Job-related health risk indicators are reported in Table 4. On average, participants reported that they worked 41.6 (±11.8) h per week. On average, they rated their job demand as 12.6 (±2.2) and job control as 24.3 (±5.18).

### 3.6. Indicators of Working Conditions by Income and Job Position

There were statistically significant differences in working conditions by income and job position. Not surprisingly, participants making less than $20,000 worked fewer hours compared to those making above $20,000 (39.8 vs. 43.5 h, *p* = 0.0001). The lowest income participants had less job control, on average (reverse coding, 25.1 vs. 23.4, *p* = 0.0001) and lower job demands (12.4 vs. 12.8, *p* = 0.0355). Administrators worked more hours compared to staff (46.4 vs. 40.6 h, *p* = 0.0001) and were less likely to report perceived job insecurity (6.03% vs. 12.5%, *p* = 0.0468). Administrators reported higher job demands (13.3 vs. 12.5, *p* = 0.0007) and better job control (21.6 vs. 24.9, *p* = 0.0001).

## 4. Discussion

This study describes health indicators on a sample of child care workers and selected contextual factors that provide insight into their work environment. Our results align with national data that child care workers are truly low wage workers. Our data also suggest that these workers exhibit many health risks such as excess weight, insufficient activity, unhealthy diet, inadequate sleep, and depressive symptoms. In addition to the hardship posed by low wages, our results confirm challenges of their working conditions such as long hours and high job demands and low job control. Also, this is the first study to explore differences in health and working conditions by household income (±$20,000) and job position (administrators vs. staff). Below, we emphasize the importance of these results in relation to existing literature, with a goal of improving future research and practice with child care workers.

The results of this study offer a valuable contribution to research into the health of child care workers, a population that has been largely ignored. Obesity (not just overweight) was an issue for the majority of child care workers in this study. Estimates from our study as well as the study by Sharma and colleagues of Head Start teachers, indicate a higher obesity prevalence among child care workers compared to the general U.S. population of adult women (66.3% and 54.5% vs. 40.4%, respectively) [12,39]. Obesity, in turn, increases risk for a wide array of chronic diseases, including cancer, heart disease, diabetes, kidney disease, and arthritis [40,41,42]. Interestingly, a greater portion of child care workers in this study appear to be sufficiently active compared to the general US population (27.8% vs. 10.7%, when applying the same cut points) [43]. These child care workers reported dietary intake and sleep patterns that are similar to the general population, but again behaviors fall short of national recommendations for overall health. For example, child care workers in our study reported eating fruits and vegetables an average of 2.6 times per day, which is similar to the average of 2.7 servings consumed by adults nationwide [44]; however, both groups fall short of the 3.5–4.5 cups recommended for women [37]. Similarly, child care workers in this study reported 6.4 h of sleep per night, which is slightly less than 6.9 h of sleep per night that most US adults report; however, both are slightly below national recommendations of 7–8 h per night [38].

An alarming 36.1% of participants in this study reported CES-D depression scores at or above the criteria for clinical depression, which far exceeds the national rate of depression for Americans (7.6%) and the rate among women between 40–59 years old (12.3%) [45]. This finding corroborates previous research demonstrating elevated levels of depressive symptoms among child care workers, including one study that found depressive symptoms among a nearly a quarter (24%) of child care staff in Head Start programs in Pennsylvania [46,47]. Depression and obesity often co-occur, so that our results warrant further investigation into the reasons why child care workers have high rates of both conditions, and, to explore effective ways of reducing their incidence and unhealthy impacts.

Our results also indicate that the lowest paid childcare workers are more likely to report multiple unhealthy behaviors. For example, they are more likely to be current smokers, drink more sweetened beverages, and report higher depressive symptoms. One potential explanation for this pattern of unhealthy behaviors is that lower wage workers are using food, beverages, or cigarettes as a way to cope with challenging work and/or financial conditions. While we cannot be certain of this explanation, we know that these behavioral choices contribute to poor health [48,49,50], and there is evidence from the literature to suggest that lower income populations are more likely to engage in these behaviors [51,52,53]. Future examinations of child care worker health behaviors would benefit from qualitative research that examines how and why these individuals are more likely to smoke or drink sweetened beverages. Then, the next generation of interventions could be tailored to the expressed needs of these individuals. For example, stress management may be a critical component of dietary and/or smoking interventions. And, interventions may need to address the underlying issues related to financial strain, either by offering assistance with finance management strategies and/or advocating for living wages.

In addition to the numerous health issues faced by child care workers, our study also highlights their challenging working conditions, including differences by job position. Consistent with existing literature, our study should serve as a call to action for addressing the child care work environment and its impact on workers’ stress and well-being [13]. Our results indicate that administrators report higher demands and higher job control than staff. We may want to investigate ways to increase the job control of the lowest income childcare workers, which are typically staff. A qualitative study by Faulkner and colleagues with home-based and center-based child care workers found that common stressors were parental interactions, caregiving, and the failure of public perception to see child care as a profession [54]. Child care workers also reported sleep disruptions (e.g., dreaming about children/work), and physical exhaustion. Child care work can be challenging, especially for administrators, who are the key gatekeepers to the child care setting. Although new interventions could be helpful, researchers should consider the readiness and capacity of child care administrators and staff when developing interventions so as not to add unnecessary burden to workers as part of well-intentioned initiatives.

Our study offers many lessons to help inform future child care-based interventions. Findings emphasize the importance of child care as a setting through which to target health initiatives, especially for those wanting to intervene with low-income women. Like other low wage earners, child care workers experience many risks to their health and well-being. These risks sometimes affect child care workers differently, based on their income and job position. Thus, future efforts to improve the health of child care workers will benefit from multi-level interventions that not only promote healthy behaviors, but also address underlying and interconnected issues related to living wages, health care benefits, and working conditions. Child care workers would also benefit from a coordinated approach to health that not only addresses physical inactivity and dietary behaviors, but also stress management and healthy coping skills. Additionally, it is not enough to focus only on the child care worker, we must address the directors, supervisors, and conditions under which these individuals operate, e.g., the entire work environment. Since child care centers are considered small businesses, we know that these organizations are less likely to provide worksite wellness and health promotion programs, policies, and environmental supports than larger employers [55,56,57]. With more than one million child care workers nationwide, many of whom are women, we need to build the evidence base for effective interventions that are tailored to this important segment of the workforce.

We acknowledge several strengths and limitations of this study. A major strength of this study is its contribution to what is currently a very limited body of literature on the health of child care workers. Our study is unique in that it includes data on the child care worker, her health status and health behaviors, and her working conditions. Another strength is the use of objective measures of several physical health indicators (e.g., physical activity, weight, waist circumference, blood pressure). Study limitations are related to cross-sectional data, possible self-selection bias, self-report bias, and unmeasured factors that may impact our findings. Specifically, because our data are cross-sectional at baseline, we cannot establish temporality of our results. We expect to be able to explore changes over time in the larger study which will have multiple measures over time. Another limitation is that we cannot generalize our findings beyond the sample of child care workers in North Carolina due to potential selection bias both at the center and participant levels. Although we cannot be certain of the impact of the bias, it is plausible that volunteers willing to participate in the worksite wellness intervention trial may be healthier than who do not participate. However, upon comparing characteristics of our final sample to the workforce in North Carolina using data from the 2015 Child Care Workforce Survey [58] we found that they appear to be similar in terms of income, education, and quality rating. While we used mostly self-report data which introduces another source of bias, we used primarily well-established instruments with sound psychometric evidence whenever available. In addition, our results may be influenced by unmeasured factors. For example, we did not measure years of work experience, but we know that in North Carolina, directors are in their positions, on average, for 6.4 years, teachers for 3.6 years, and teachers assistants for 2.5 years [58]. So the results we report based on income and/or job position may be better understood if we knew length of time in child care. It is also likely that the relationships we discovered are due to an overlap between income and job position. We did not collect data on the relationship between administrators and staff which can contribute to high job strain if these relationships are negative or otherwise unsupportive. And, we have no information about the stress that child care workers may be under at home which will also likely influence health [59,60]. These are several examples of unmeasured variables that could provide additional insights about our results and future studies should consider.

The next generation of research might benefit from considering integrated interventions that address both health promotion and occupational health and safety. The National Institute of Occupational Safety and Health (NIOSH) is advocating for “Total Worker Health” interventions that may be particularly appropriate for this group of workers and workplaces [61,62]. Moreover, future research should include mixed methods studies that would explore reasons why child care workers practice unhealthy behaviors or rate work experiences as high demand/low control; as well as work with center administrators to determine who can best influence the policies and practices in place at child care centers. Promoting the health of child care workers at the workplace with health programs, policies, and environmental supports, along with higher wages, is critical.

## 5. Conclusions

Child care centers are located in all states and employ over 1.3 million workers nationally [4]. While our results are specific to those who participated in this study, these findings provide useful insights for the larger population of child care workers nationally. These results represent an important call to action for researchers, policy makers, and community leaders who can advocate for living wages for these important members of the workforce, and plan interventions to improve the health and well-being of child care workers in the context of their everyday work conditions.

## Figures and Tables

**Table 1:** Participant demographics.

Demographic	*N* (%)	Mean (SD)
Age (years)		39.9 (13.0)
Sex		
Female	674 (100.0)	
Race		
Caucasian	244 (37.1)	
African American	364 (55.4)	
Asian	7 (1.1)	
American Indian/Alaskan Native	13 (2.0)	
Other	4 (0.6)	
Mixed	25 (3.8)	
Ethnicity		
Hispanic	36 (5.3)	
Highest level of education		
High school diploma/GED	82 (12.2)	
Some college	267 (39.6)	
Associate’s degree	163 (24.2)	
Bachelor’s degree	136 (20.2)	
Graduate, MS, or higher	26 (3.9)	
Married/living with a partner	338 (50.2)	
# in household		3.28 (1.70)
Household income (annual)		
<$10 K	62 (9.2)	
$10–15 K	106 (15.7)	
$15–20 K	115 (17.1)	
$20–25 K	78 (11.6)	
$25–35 K	85 (12.6)	
$35–50 K	77 (11.4)	
$50–75 K	41 (6.1)	
>$75 K	36 (5.3)	
prefer not to answer	74 (11.0)	
Health insured	506 (75.1)	

**Table 2:** Childcare center descriptors.

Descriptor	*N* (%)	Mean (SD)
Years in operation		17.4 (11.3)
Hours of operation		13.1 (3.36)
Enrollment fee (dollars/week) *		140.3 (19.5)
Star rating		4.30 (0.67)
Size		
# of children		56.3 (32.5)
# of employees		12.1 (8.59)
Employee role		
Administrator	118 (17.5)	
Staff	556 (82.5)	
Faith-based	20 (27.0)	
Early Head Start	1 (1.4)	
Privately owned	49 (68.1)	
Accepts subsidies	72 (97.3)	
CACFP participation	61 (84.7)	
NAEYC accredited	11 (15.3)	

* Average enrollment fee for a 3–5 year old.

**Table 3:** Physical and mental health risk indicators.

Variable	All		Below $20 K		Above $20 K		*p*-Value
	*N* (%)	Mean (SD)	*N* (%)	Mean (SD)	*N* (%)	Mean (SD)	
BMI (kg/m^2^)		34.5 (9.01)		34.8 (9.24)		34.3 (8.80)	0.5194
Underweight	5 (0.8)		2 (0.7)		3 (1.0)		
Normal Weight	64 (10.7)		29 (10.3)		35 (11.0)		
Overweight	133 (22.2)		58 (20.5)		75 (23.7)		
Obese	398 (66.3)		194 (68.6)		204 (64.4)		
Body weight (kg)		90.4 (25.1)		91.1 (25.7)		89.8 (24.6)	
Waist circumference (cm)		106.5 (18.2)		106.9 (18.8)		106.2 (17.7)	0.6024
Heart rate (bpm)		77.6 (11.9)		78.2 (11.4)		77.1 (12.4)	0.2829
Mean arterial pressure		94.6 (14.0)		94.2 (15.1)		94.9 (13.0)	0.5725
Systolic blood pressure (mm/Hg)		122.9 (19.5)		122.2 (20.9)		123.5 (18.3)	
Diastolic blood pressure (mm/Hg)		80.4 (12.6)		80.1 (13.7)		80.6 (11.8)	
MVPA (min/week)		122 (104)		126 (95.0)		120 (110)	0.5220
Meeting physical activity recommendations	142 (27.8)		67 (29.8)		75 (26.3)		0.3864
Sedentary time (min/day)		481 (72.4)		468 (73.1)		491 (70.5)	0.0006 *
Current cigarette smoker	93 (15.6)		56 (19.9)		37 (11.7)		0.0057 *
Ever used e-cigarettes	59 (9.9)		30 (10.7)		29 (9.2)		0.5402
Fruit and vegetable consumption (times/day)		2.58 (1.72)		2.52 (1.83)		2.64 (1.61)	0.4156
Sweetened beverage consumption (times/day)		1.71 (1.82)		1.93 (2.06)		1.51 (1.56)	0.0057 *
Sleep duration (hours/night)		6.37 (1.35)		6.34 (1.39)		6.40 (1.31)	0.6399
Perceived level of distress		4.02 (2.78)		4.10 (2.82)		3.94 (2.75)	0.4652
CES-D score		13.9 (9.17)		15.5 (9.95)		12.6 (8.22)	0.0002 *
CES-D ≥ 16	215 (36.1)		116 (41.6)		99 (31.3)		0.0094 *

* *p* < 0.05.

**Table 4:** Job-related health risk indicators.

Variable	All		Administrator		Staff		*p*-Value
	*N* (%)	Mean (SD)	*N* (%)	Mean (SD)	*N* (%)	Mean (SD)	
Hours worked (hours/week)		41.6 (11.8)		46.4 (12.2)		40.6 (11.4)	<0.0001 *
Perceived job insecurity	76 (11.4)		7 (6.03)		69 (12.5)		0.0468
Job demands (range 5–20)		12.6 (2.19)		13.3 (2.41)		12.5 (2.12)	0.0007 *
Job control (range 12–48)		24.3 (5.18)		21.6 (5.04)		24.9 (5.03)	<0.0001 *
Skill discretion (range 6–24)		12.2 (2.31)		11.3 (1.99)		12.4 (2.33)	<0.0001 *
Decision authority (range 6–24)		12.1 (3.45)		10.2 (3.51)		12.5 (3.32)	<0.0001 *

* *p* < 0.05.
